# Listen to the Whispers before They Become Screams: Addressing Black Maternal Morbidity and Mortality in the United States

**DOI:** 10.3390/healthcare11030438

**Published:** 2023-02-03

**Authors:** Anuli Njoku, Marian Evans, Lillian Nimo-Sefah, Jonell Bailey

**Affiliations:** Department of Public Health, College of Health and Human Services, Southern Connecticut State University, 493 Fitch Street, New Haven, CT 06515, USA

**Keywords:** black maternal mortality, morbidity, social determinants of health, weathering framework, health disparities, race/ethnicity, COVID-19, racism, intersectionality, pregnancy

## Abstract

Black women in the United States (U.S.) disproportionately experience adverse pregnancy outcomes, including maternal mortality, compared to women of other racial and ethnic groups. Historical legacies of institutionalized racism and bias in medicine compound this problem. The disproportionate impact of COVID-19 on communities of color may further worsen existing racial disparities in maternal morbidity and mortality. This paper discusses structural and social determinants of racial disparities with a focus on the Black maternal mortality crisis in the United States. We explore how structural racism contributes to a greater risk of adverse obstetric outcomes among Black women in the U.S. We also propose public health, healthcare systems, and community-engaged approaches to decrease racial disparities in maternal morbidity and mortality.

## 1. Introduction

The Centers for Disease Control and Prevention (CDC) reports that 50,000 women in the United States (U.S.) suffer from pregnancy complications annually, but that Black women are at least three times more likely to die due to a pregnancy-related cause when compared to White women [1,2]. The estimated maternal mortality rate in 2019 was 20.1 and, in 2020, was 23.8 per 100,000 births which represents about 861 maternal deaths. For Black women, that rate is about 55.3 per 100,000 live births, representing an estimated 1800 maternal deaths, the highest amongst any racial group; this is a number that has continued to increase over the past few years [3,4]. While each mortality or morbidity circumstance is different, the leading causal factors associated with maternal mortality and morbidity in the U.S. include hypertensive disorders of pregnancy, thrombotic pulmonary embolism, hemorrhage, infection, cardiovascular conditions, cardiomyopathy, and non-cardiovascular medical conditions [5]. While predisposition to underlying health conditions such as hypertension, cardiovascular disease, diabetes, and obesity plays a role in racial disparities in pregnancy-related deaths and other adverse pregnancy outcomes, when these medical conditions are not present, racial disparities persist.

More recent studies have shown that social factors such as historical exposure to racial trauma, discrimination, and marginalization; systemic barriers such as systematic racism and implicit bias within the healthcare system; the possibility of being uninsured; reduced access to reproductive healthcare services; and socioeconomic factors also contribute to pregnancy complications for Black women and have to be given consideration [2,5,6,7,8]. These social determinants of health show that poor maternal outcomes for Black individuals are caused by factors of racism that are embedded in healthcare and affect marginalized groups of individuals disproportionately. Based on socioeconomic status, race, age, and other identifying factors, the health disparities amongst individuals in communities that lack resources and education is exacerbated and continues to expand the gap in access to equitable health [9]. The history of racism within healthcare must be understood to dismantle institutionalized racism in healthcare systems and to create policies that protect Black women. Social and systemic changes are imperative to reduce Black maternal morbidity and mortality. Therefore, the stark differences in reproductive health outcomes for Black women necessitate an increased focus on the intersectional roles of racism, discrimination, and other social determinants of health in influencing disease and mortality risk.

Within the 21st century, healthcare has seen drastic shifts, especially with the ongoing COVID-19 pandemic. Research has shown that maternal mortality increased by 33% after the start of the pandemic and that late maternal deaths increased by 41% [10]. Moreover, the percentage of maternal deaths was even higher among Black and Hispanic women during the early part of the pandemic period, with increases in underlying cause-of-death codes for conditions such as other viral diseases (2374.7%), diseases of the respiratory system (117.7%), and diseases of the circulatory system (72.1%) [10]. COVID-19 represents a major social stressor for all at many levels but especially regarding maternal health [11]. While pregnant women overall were not found to have a higher risk for COVID-19 infection, women of color that were infected often experienced more adverse outcomes, as well as faced disproportionately adverse socioeconomic consequences. Maternal health was impacted due to the current COVID-19 pandemic both explicitly due to a life-threatening infection and indirectly due to the necessary changes in healthcare for infection control purposes. Isolation and quarantine as age-old infectious disease prevention protocols were instituted and strictly enforced. Some examples of healthcare COVID-19 protocols and hospital instituted labor and delivery changes consisted of less familial support available in the delivery room, long wait times, provider shortages, and overall hesitancy to seek prenatal care, which affected pregnant women’s mental health drastically, with studies showing increases in depression and anxiety [12]. Overall, during the COVID-19 pandemic, pregnant women experienced the stress of social changes in their jobs, families, and the fear of how to keep themselves healthy and safe.

The purpose of this paper is to discuss structural and social determinants of Black maternal mortality in the United States This perspective paper will also propose some public health, health systems, and community-engaged approaches that reduce racial disparities in maternal mortality and morbidity while striving to achieve equity in maternal health outcomes among Black women in the United States.

### Theoretical Framework

**Figure 1:**
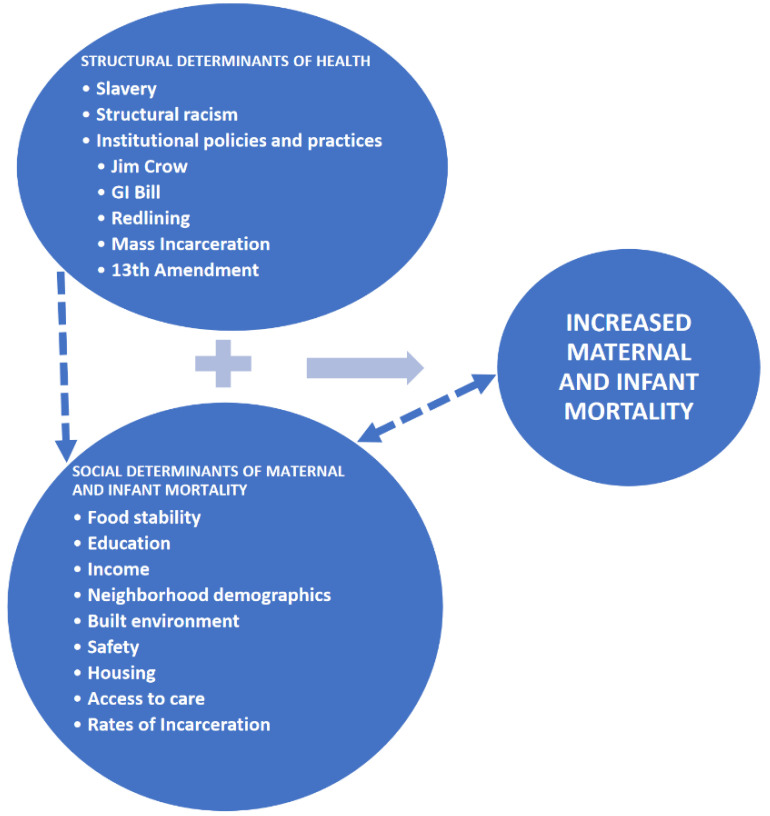
Application of Theoretical Framework on Structural and Social Determinants of Maternal and Infant Mortality in the United States.

The effects of racism in our society erode Black people’s health in a multitude of intersectional ways and dimensions. One of the theoretical frameworks that guide this paper is the “Weathering” framework. This framework’s foundation was originally rooted in maternal health, morbidity, and mortality and directly challenges the historical societal narratives of teen pregnancy, fertility peaks, and birth timing for African American women. In 1992, Dr. Arline Geronimus hypothesized correctly that the effects of racism in our society cause “premature biological aging”, hence the “weathering” in African American women, which has a direct effect on infant and maternal morbidity and mortality, and overall birth outcomes [13]. This weathering creates an overall “general health vulnerability” [14,15], which is a consequence of all levels of racism in the United States. Data reveal that there are increasingly poor pregnancy and birth outcomes as young Black women delayed fertility past their late teens, while this was not seen in White females. Since Dr. Geronimus’ seminal article, multiple quantitative biological marker studies have borne out the detrimental racial effects of a concept entitled, “allostatic load” or chronic stress [14,15,16]. Wakeel (2021) and colleagues further expanded the weathering theoretical framework by synthesizing the intersectionality of an older socioecological model (SEM) and the social determinants of health (SDOH) considering COVID-19 [11]. A “stressful life event” such as COVID-19 exacerbates all other social determinants of health [11]. Furthermore, we propose a theoretical framework adapted from Roach’s Restoring Our Own Through Transformation (ROOTT) Theoretical Framework [17], which explores how structural and social determinants impact maternal and infant mortality in the United States (Figure 1). In this conceptual framework (Figure 1), structural determinants of health are characterized by factors such as slavery, structural racism, and institutional policies and practices such as Jim Crow laws, the G.I. Bill (the Servicemen’s Readjustment Act of 1944), redlining, mass incarceration, and the 13th Amendment. These structural determinants of health shape social determinants of maternal and infant mortality, with this process indicated by the connection of dashed lines. These social determinants of health include food stability, education, income, built environment, neighborhood demographics, safety, housing, access to care, and incarceration. Structural determinants of health and social determinants interact in multiple and interrelated ways to influence increased maternal and infant mortality in the United States and work to exacerbate disparities in health outcomes. Furthermore, social determinants of health shape and are influenced by increased maternal and infant mortality. It is also important to consider intersectionality as an analytical framework that explores the unique experiences of Black women encountered at the intersections of race, class, and gender [18].

## 2. Social Determinants of Health

Social determinants of health are non-medical factors that affect health outcomes and include biology, individual behavior, socioeconomic status, physical and social environment, support, racism, discrimination, access to affordable health services, and legislative policies [19,20]. Social determinants of health can occur across multiple levels for women and children, intersecting across several domains of influence including biological, behavioral, physical, and sociocultural environments and the healthcare system [21]. Social determinants of health are primarily responsible for health inequities, or avoidable and unfair differences in health status between diverse groups of people within the same country and between countries [20,22]. Mitigating the root determinants of health to reduce health inequities is vital because health is a fundamental human right, and the inability to prevent inequities results in health disparities [20,22]. Various social determinants play a key role in producing and maintaining adverse maternal outcomes in the United States, with empirical studies showing that race and ethnicity, education, and insurance (including access to prenatal care) contribute to the establishment and continuation of pregnancy-related mortality and severe maternal morbidity risk [23,24,25]. Place-based factors such as neighborhood conditions, access to quality healthcare and amenities, environmental exposures (heavy metal exposure, pesticides, pollution, and traffic), and residential segregation have been associated with unfavorable birth outcomes among Black and Hispanic women and increased maternal morbidity and mortality [7,26,27,28].

Structural and social determinants can be further explored to demonstrate their link to racial disparities in maternal and infant mortality. The historical legacy of slavery is linked to Black maternal and infant health, and contemporary maternal and infant mortality [29]. Maternal and infant health disparities are rooted in the institution of slavery, which commodified enslaved Black women’s childbearing and empowered physicians to authorize the interests of slaveowners [29,30]. Moreover, while overall infant death rates have declined since the 19th century, the disparity in death rates between Black and White infants is greater today than it was under prewar slavery [31,32]. Currently, the stressor of structural racism permeates and reverberates within the lives of Black women and their children [29]. Racism’s effects are seen at the genetic and physiological levels and reveal persistent maternal and infant death disparities [15]. For example, conditions such as hypertension have been associated with the stress of inhabiting a racist society and can further exacerbate disparities in pregnancy-related complications such as pre-eclampsia. Such factors prompt critical exploration into the underlying initiators of such disparities [33].

Racism as a social construct has been identified as a persistent population health emergency and a fundamental cause of disease both in the U.S. and globally. Studies have comprehensively illustrated the multidimensional associations between racism at the cultural (e.g., derogatory, and exclusionary stereotypes), interpersonal (e.g., macroaggressions rooted in implicit bias and decreased likelihood of receiving patient-centered care), and structural levels (e.g., laws, regulations, and policies that steadily lead to reduced access to opportunities and services based on race), and health outcomes among Black persons [34,35,36,37,38,39,40,41,42,43,44,45,46,47,48,49]. Experiences of racism are also apparent across the sexual and reproductive health lifespan. Structural racism can impact Black women’s use of reproductive services and can sustain reproductive healthcare disparities; structural racism can influence access to care through the ability to attain timely services, the use of healthcare services, and experiences with care related to interactions with the healthcare system [8]. Notably, there can be an intergenerational transmission of the stress that stems from cumulative exposure to interpersonal racism, illustrated by the fact that both stressful life events and perceived stress, before, during, and after pregnancy, have been associated with unfavorable pregnancy and childbirth outcomes; effects include pregnancy complications, preterm birth, and low birth weight, which resultantly have significant and multifaceted implications for longstanding maternal and child health outcomes [6,40,41,42,43]. Moreover, the intergenerational transmission or risk associated with structural racism is particularly manifested in the greater risk of adverse obstetric outcomes and increased infant mortality rates in U.S. Black communities [26,27].

Regarding policies and practices, Jim Crow laws legalized segregation in Southern U.S. states from the 1870s through the mid-1960s and exposed Black persons to noxious social, economic, and physical conditions that could influence access to care [44]. Being born in a Jim Crow state has been shown to influence population health indicators such as infant death, and the health effects of state-sanctioned racism in the 1960s to this day can be seen in infant death inequities. Specifically, Black infant death rates were almost two times higher in Jim Crow states than non-Jim Crow states from 1960 to 1964 [45]. It is known that early-life traumatic exposures can influence the risk of any type of health issue. Understanding determinants of health inequities within and across generations involves recognizing that people carry the history of a country within their bodies [45,46].

In another example of structural determinants of health, President Roosevelt’s race-neutral G.I. Bill, a law that went into effect in 1944 and provided a range of benefits for qualifying returning World War II veterans and their families, had state-controlled oppositions that kept many Black veterans from acquiring its full benefits. Resultantly, Black veterans and their families were deprived of their fair portion of the multigenerational, enhancing effect of home ownership, educational, and economic security that the G.I. bill bestowed on most White veterans, their children, and their grandchildren [47]. Redlining, or government-sponsored disinvestment in non-White neighborhoods, to be explained further in this paper, is a structural determinant of adverse maternal and infant health outcomes [9].

The mass incarceration of Black persons in the U.S. is largely the result of institutional policies in U.S. police and judicial systems, including aggressive enforcement of low-level drug offenses and mandatory punitive sentencing laws that excessively affect Black persons [48]. Furthermore, incarceration in the family can play a crucial role in affecting Black, Indigenous, and people of color (BIPOC) women’s life, including during a pandemic. Mass incarceration can have important maternal and child health considerations such as the availability of sufficient social support during pregnancy and delivery and can adversely impact BIPOC populations during the COVID-19 pandemic [49]. Additionally, Black women may have to contend with the increased likelihood of having a partner suffer an injurious or fatal interaction with law enforcement due to the persistent issue of police brutality against Blacks in the United States [50].

In another example, while the 13th Amendment abolished slavery by the start of the twentieth century, the interstate slave trade was still legal under U.S. law. Children of the enslaved were enslaved by birthright, Black women’s bodies were commodified as their ability to reproduce was of utmost importance, and enslaved women’s reproductive lives garnered increasing attention from White physicians. Lastly, we believe that these social conditions are intersectional and interlaced and have reinforced lasting effects that can be manifested in the bodies of Black women. These issues highlight various concerns related to reproductive justice, human rights, and birthing autonomy [51].

## 3. Black Maternal Morbidity and Mortality

### 3.1. Contributing Factors

**Figure 2:**
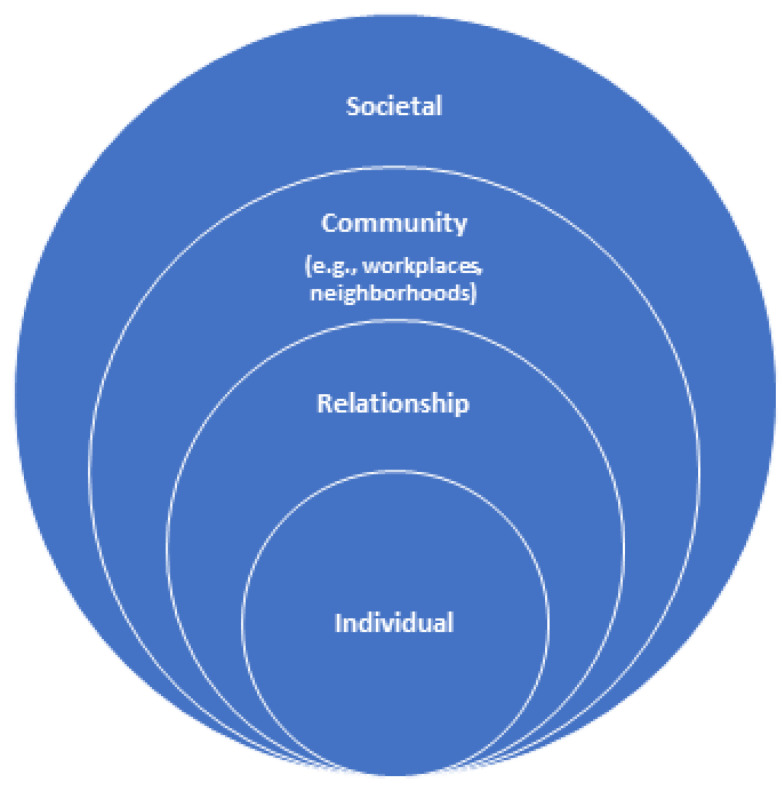
Socioecological Model for Prevention.

The Weathering Framework is germane to this paper as it combines the Socioecological Model and Social Determinants of Health. The socioecological model shows that behavior has multiple levels of influence; the model helps examine factors that influence a specific behavior (See Figure 2). The CDC considers this model as a framework for prevention [52]. The model contains five levels: individual, interpersonal, organizational, community, and societal, as recreated in this paper (See Figure 2). Following this socioecological model for prevention, contributing factors toward racial disparities in maternal morbidity and mortality can be examined (Figure 2). Through this lens, a range of factors that put Black women at risk can be understood. Racism is a social construct, root cause, and determinant of maternal morbidity and mortality. Historically, when social support is increased in the U.S., maternal morbidity, and mortality as an indicator of a society’s well-being are improved. This is due to the intersectionality of the following levels of influence. Furthermore, the relationship between factors at one level of influence that can impact factors at another level can be studied.

#### 3.1.1. Individual Factors

Individual contributing factors describe biological and personal traits that cause certain behaviors. Examples include biological and genetic factors, beliefs, attitudes, education, stress response, and even coping skills. One biological factor is age, which is a nonmodifiable risk factor. Data show that the maternal mortality rate for Black women between ages 30 and 34 is over four times higher than the rate for White women [53]. Existing health conditions such as cardiovascular disease, diabetes, and high blood pressure can be detrimental to a pregnant woman. These health problems are preventable if quality care is given and access to education is available. 

Black women have a maternal mortality rate of 2.9 times that of White women in the United States [11]. For several years, Black women have been ignored and dismissed by medical providers in the United States. Even as medicine progresses, racial disparities persist [54]. Black women continue to be failed by blatant negligence and ignorance. As a result, Black women may lose the fight to be heard by their providers. 

Individual traits of women can either help or work against them. For example, an individual contributing factor is education and knowledge. Unfortunately, many women are not well informed about the importance of preconception health, their specific pregnancy milestones, or even frequently occurring conditions. As a result, this makes them more susceptible to being ignored. They may not be capable of advocating for themselves and may reluctantly be mistreated because they are unaware. On the other hand, a very educated woman who is more knowledgeable about her pregnancy and concerns may still be misheard or ignored by medical providers because “they know best”.

Stress response and chronic stress are other types of individual contributing factors. Humans produce stress hormones when stressed. However, when stressed all the time, the body will have elevated levels of stress hormones. Elevated stress relates to physical and mental health conditions that can lead to death [55]. Studies have shown that just being a woman is a stressor in our society. Being a Black woman causes a “double pressure” influence on women [56]. The pressure of being a Black woman is immense and can have effects on a pregnancy [55]. Different types of stress that can occur during pregnancy include negative life events, catastrophic events, long-lasting stress, chronic stress, and racism [57]. Racism is a large contributor to stress, and it is evident through data that shows African American women in the United States deliver a higher rate of premature and low-birthweight babies than their counterparts [58]. Attitudes and beliefs are important individual factors. There is compelling data to suggest that when Black Women and babies are dying due to pregnancy complications or possible negligence from providers and the healthcare system, this puts Black women on edge [54]. They enter situations with preconceived notions about how they will be treated. This in turn affects their expectations of care and whether they feel empowered to speak up about the care they are receiving. Other individual factors such as alcohol intake, being a current or former smoker, nutritional status (e.g., chronic energy deficiency vs. good nutrition), and occupational status (e.g., whether a person is working or not) have been shown to increase a woman’s risk for maternal morbidity and mortality [59,60,61]. Living in a disadvantaged neighborhood has been linked to a higher allostatic load in African American women at risk for obesity and related chronic diseases [62,63,64]. Moreover, neighborhood disadvantage in the context of allostatic load can influence individual health behaviors such as alcohol and tobacco consumption, diet, and exercise [65]. Economic and psychosocial factors have been shown to explain 36–42% of racial and ethnic inequalities in postpartum allostatic load [66]. Therefore, structural factors that shape social determinants such as neighborhood features can also influence individual behaviors that place Black women at increased risk for maternal morbidity and mortality.

#### 3.1.2. Interpersonal Factors

Interpersonal contributing factors describe relationships. An individual’s immediate social circle (friends, spouse, and family members) influence their behavior and affects their experiences. During pregnancy, several relationships will be formed. Examples include doctor–patient relationships as well as family and peer relationships. International and national studies commonly find that preventable maternal deaths are due to provider factors. These include ignoring and withholding diagnoses, lack of appropriate referrals, and poor documentation and communication [67]. Doctor–patient relationships are especially important from the start of a pregnancy. However, several studies have shown negative maternal and child health providers’ attitudes and behaviors affect patients’ well-being, satisfaction with care, and care-seeking [68]. Trust is a crucial factor in relationships. Social roles and social isolation must be considered as well in these contributing factors. History of mistreatment can affect the support received from family and friends. This occurrence could influence an expecting mother’s thoughts or behaviors toward her pregnancy journey. 

Social isolation is an interpersonal contributing factor, and it can be damaging to health. COVID-19 further perpetuates its occurrence. COVID-19 negatively impacted the social life of pregnant women. Loss of social support can affect how a mother will advocate for herself. Women reported that the pandemic created fear and anxiety among pregnant patients because of limited doctor visits and financial issues. Women reported fear and anxiety concerning poor perinatal services [69]. There can also be a lack of advocacy on a woman’s behalf from family and friends. Lack of this support causes anxiety about giving birth as well. A study showed that women felt that COVID-19 separated them from their families and put a strain on interpersonal relationships. An increased fear of catching the new disease weakened support systems and increased dependency on providers that mothers did not feel close to [69]. Lack of support during the delivery or labor process isolates mothers and contributes to their concerns being disregarded.

#### 3.1.3. Organizational Factors

Systemic inequalities are often first seen at organizational levels. Organizational contributing factors include schooling and educational opportunities, community services, and access to resources. Quality access to health care, especially in terms of physical proximity, can be a challenge for individuals in certain neighborhoods and communities. Regardless of race, mothers living in impoverished areas are prone to die from maternal mortality [70]. Implicit bias among providers and staff highlights the importance of cultural competency, shared decision-making, and acknowledgment of personal biases to address disparities in care [67]. Furthermore, implicit bias can lead to racial disparities in maternal morbidity and mortality in the U.S. [71]. Implicit bias and discrimination within the healthcare system can be revealed in the dismissal of Black women’s symptoms and concerns, which can elucidate the poor outcomes even for Black women with higher levels of education and income [72]. Provider actions and their interactions with patients are strongly linked to racial disparities in the endurance of trauma during childbirth. Thirty percent of Black and Hispanic women in the United States who delivered in a hospital reported provider mistreatment compared to twenty-one percent of White women [73]. In a survey on maternity care among women in California, Black women were ten times more likely to report unfair treatment and discrimination from maternity care providers when compared to White women [74]. Community-based care could be used as an effective method of providing more access to care and resources to mitigate maternal mortality. Community-based care can include home-based care by more certified midwives, community-operated clinics, and health campaigns [75]. These efforts should include reinforcement of preconception and postpartum care to target racial disparities in maternal morbidity and mortality. Furthermore, multidisciplinary quality care initiatives that partner with communities can enhance the quality of care and reduce disparities [67]. Social mobilization, health promotion and education, and advocacy are needed to promote health, and to give people the knowledge and skills needed to improve their health or to advocate for themselves.

#### 3.1.4. Community Factors

Relationships will occur in community settings (i.e., schools, workplaces, and neighborhoods) and can become a contributing factor to how maternal health is affected and the outcomes in maternal health for Black women. As mentioned at the organizational level, discrimination in one’s surroundings has a notable impact on healthcare and health outcomes. For example, a recent study in Chicago showed a relationship between racial residential segregation and the presence of hypertensive disorder in Black pregnant women living in impoverished neighborhoods [76]. This further supports that there is a correlation between health and racial residential segregation. These urban communities with large Black populations tend to be impoverished and underfunded and lack adequate resources such as stable housing and suitable transportation, which are fundamental causes of poor physical health and further disadvantage the people who live there, which include Black pregnant women. This is caused by instances of systematic racism, which cause social and structural determinants of maternal and infant mortality in the United States.

There are many practices within communities that originate from structural racism. For example, redlining (defined as home mortgage denial based on race and government-backed disinvestment in non-White neighborhoods) was created as an oppressive form of housing historically and underserved individuals living in these areas. This created unhealthy habits within a community such as less emphasis on physical activity. This causes health disadvantages for those who are in this community such as financial barriers to care, access to quality healthcare, lack of education, and a shortage of primary care providers [77]. Racial and ethnic disparities in postpartum care before and after the COVID-19 pandemic also influence maternal mortality and severe morbidity among Black women [78,79]. The postpartum period is a crucial time for women to recover from childbirth and to adjust to several biological, social, and psychological transitions [80]. The postponement and absence of prenatal care, which was more likely to occur among Black and Hispanic women even before the COVID-19 pandemic [81,82], can obstruct prevention of maternal mortality and increase the likelihood of emergency room visits, childbirth complications, postpartum depression, and unmet postpartum care needs [83,84]. One study showed that, when compared to White women, Black women had an increased probability of not scheduling postpartum care and the slowest reduction in postpartum care canceling rate during the COVID-19 pandemic [85]. Black women have also been at increased risk for worries about prenatal care [79] and postpartum stress during the COVID-19 pandemic [86]. It is important to acknowledge the role of structural inequities and intersectional vulnerabilities that increase risks for unfavorable maternal health outcomes and fuel health disparities [85]. One way to address this contributing factor is to invest in primary care within a community that can tend to diverse women that differ in race, age, and socioeconomic status in a variety of settings. Midwifery, doulas, maternity centers, nurse practitioners, and clinical settings can greatly impact maternal health in Black communities [87].

#### 3.1.5. Societal Factors

The goal of reducing Black maternal mortality involves a vast approach that includes the patient, provider, and public health policies. Concepts such as structural racism have been proven to be the main reason for the disparities that occur across the different levels of influence. Structural racism is a large and deep-rooted force in society that can factor into health care. For example, there may be a racial bias that is present among certain healthcare professionals, which can cause hindrance to the care and treatment of Black pregnant patients. These patterns cause mistrust among Black patients and their providers as well as the medical community at large [8].

One of the structural determinants of maternal health that is linked to U.S. health disadvantages for individuals is the suffering of financial barriers to care [24]. This results in the lack of primary care providers, which creates poor, insufficient care for individuals. With healthcare facilities understaffed and overworked, many patients have a feeling of being overlooked and healthcare professionals are reporting burnout. There is a shortage of obstetricians, nurse midwives, and well-women nurses that serve in low-income, racially, and ethnically diverse communities. The lack of medical personnel can drastically affect the outcome of a pregnancy. Another structural determinant at the societal level is education [9,47]. Structural racism can also manifest in historical and contemporary practices such as redlining and segregation, which can hinder access to educational resources and opportunities and perpetuate intergenerational poverty due to less access to parental materials [7,9,36,39]. Additionally, the intergenerational transmission of risk attributed to structural racism is most concretely illustrated through the increased risk of adverse obstetric outcomes and higher infant mortality rates in African American communities. For example, research has indicated that African American women exposed to residential segregation are more likely to experience adverse birth outcomes, even after controlling for individual and neighborhood-level poverty [26,27]. While socioeconomic inequities rooted in structural racism and discrimination are primary drivers in racially disparate maternal health outcomes, differences in insurance coverage also play a role [88]. The Medicaid coverage gap, which can occur when individuals of low income lack a path to affordable coverage due to living in one of 12 U.S. states that have refused to expand Medicaid, is rooted in structural racism, and affects maternal morbidity and mortality [89]. Medicaid covers over 40 percent of U.S. births and 65 percent of births to Black mothers; almost 30% of Black women of reproductive age are in the Medicaid coverage gap, which limits their access to quality preconception and prenatal services and prospects of a safer pregnancy and birth for a parent and baby [89].

## 4. Discussion

An exploration of the factors that contribute to racial disparities in maternal morbidity and mortality among Black women in the U.S. calls for public health, the healthcare system, and community-engaged approaches to achieve equity in maternal health outcomes. These types of barriers could be addressed by targeting the underlying social determinants that fuel the rates of Black maternal morbidity and mortality and by incorporating policy and educational modifications to the healthcare system and industries that supply the healthcare system. We propose the strategies below to reduce racial disparities in maternal morbidity and mortality.

### 4.1. Enhance Curriculum and Diversify the Workforce to Address Implicit Bias and to Improve Cultural Humility

Evidence strongly supports the impact that structural racism continues to have on our healthcare sector [11]. Diversifying the medical workforce is imperative to help with this crisis. Currently, although Black individuals make up 13% of the population, they comprise just about 5% of the active physician workforce. “Black female physicians comprise even less, representing only 2% of physicians overall” [90]. This illustrates the importance of racially concordant care and encourages efforts to address implicit bias and to improve cultural humility within the healthcare workforce. Healthcare providers can use clinical resources and tools to recognize and address unconscious bias and stigma in themselves and in their offices to promote cultural awareness and health equity [77]. To remedy implicit bias across the continuum of maternal health care, hospitals and healthcare systems can train obstetric and non-obstetric care providers to build knowledge and skills on cultural humility, cultural competency, and person-centered care [87].

Medical schools and health profession programs should incorporate social determinants of health and health disparities education into the curriculum to equip students with an appreciation of cultural competence, to help them identify and address racial bias in themselves and medicine, and to clarify how health disparities can unfavorably affect both patient and healthcare system outcomes [91]. Outlining how health disparities and contributing social determinants can result in excess medical care costs, lost productivity, and premature deaths can help reduce healthcare system costs and improve the quality of care for everyone [92]. Additionally, curriculum development should consider interprofessional, collaborative efforts with other health professions disciplines to foster a multidisciplinary approach to addressing health disparities [93]. Patient education and clinical workforce training initiatives can partner with community health organizations and academic researchers to raise awareness about racial disparities in maternal and child health outcomes [94]. There should also be enhanced training and education in maternal–fetal medicine to improve the management and medical care of pregnant women to address racial disparities in maternal mortality and severe morbidity [95]. Additionally, there should be efforts to increase and diversify the perinatal workforce (e.g., doulas, certified and lay midwives, and perinatal social workers) to decrease maternal and neonatal morbidity and mortality [96].

### 4.2. Explore the Impact of Environmental and Occupational Exposures on Maternal Morbidity and Mortality

There is a need to explore the impact of disparate environmental and occupational exposures on maternal morbidity and mortality [7,26,27,28,97]. Psychosocial stressors such as police brutality can impact Black mothers’ lives when Black mothers endure a gendered racial vulnerability with their added responsibility of teaching their children to respond to police violence in the “police talk” [98,99]. Such responsibilities that stem from structural racism can cause physical manifestations of stress and psychological distress and have been associated with depressive symptoms among Black women [58,100,101,102,103]. Moreover, as previously mentioned, incarceration in the family can play an immense role in affecting BIPOC women’s life and have important maternal and child health considerations, including adversely impacting the availability of adequate support during pregnancy and childbirth among BIPOC populations [49]. It is also important to examine how structural racism and discrimination in the workplace environment can take a toll on Black mothers through manifestations such as microaggressions, increased emotional trauma, the gender pay gap, invisibility, negative stereotypes, tokenism, and isolation [101,102].

When further considering environmental impacts on maternal mortality, it is evidenced that racial and ethnic inequities in social determinants of health, such as neighborhood environment (e.g., access to healthy food, neighborhood safety, housing, air pollution, pest, and mold exposure), environmental exposures (e.g., experiences of racism, discrimination, immigration, and acculturation), socioeconomic status (e.g., income, wealth, educational attainment, and employment), housing (e.g., housing conditions such as indoor air pollution and microbial/pest allergen exposures), and health care access and quality, add to the excess burden of chronic disease incidence, prevalence, morbidity, and mortality among particular racial and ethnic groups, including Black communities. Other recommendations include safe housing and environmental justice efforts that include the reduction, remediation, and prevention of environmental lead hazards in older housing to remedy inequitable exposure of lead to Black residents, including disparate lead exposure to Black children [103]. Moreover, BIPOC and low-income communities, both in residential and workplace settings, as well as in rural and urban areas, shoulder a disproportionate burden of the harms caused by pesticides in the United States, which have maternal and child health implications. There is an increased application of pesticides in urban and low-income public housing [104]. Remediation policies are needed where women and children in these communities may be disproportionately exposed to pesticides through an increased likelihood to reside near pesticide manufacturing facilities that may possibly violate environmental laws [104].

### 4.3. Address Social Determinants of Health by Exploring the Impact of Structural Racism on Maternal Health Outcomes

There is also a need to address social determinants of racial disparities in maternal morbidity and mortality by exploring the impact of structural racism on access to factors such as quality healthcare (e.g., the effect of structural racism/historical abuses on health-seeking behaviors and confidence in the health care system), education, income and employment, and quality food. Structural racism affects health through its past and present effects on the quality of, and equal access to, key social, and environmental determinants of health. For example, the practice of redlining inhibited communities of color from acquiring residential mortgages and, accordingly, access to public transportation, supermarkets, and healthcare, contributing to the proliferation of residential segregation in the United States [35,44,105,106]. Resultantly, in U.S. communities plagued by segregation, Black persons and other racial and ethnic minority groups are more likely to live in neighborhoods with increased levels of poverty; to have reduced access to employment, credit, housing, educational, transportation, nutritional, and healthcare resources; and to live in health-inhibiting environments, compared to the White population [35,107]. Systemic racism also inhibits access to vital healthcare services, such as access to reproductive and sexual health services [8]. Therefore, there is a need to address these structural barriers and to acknowledge their role in racially disparate maternal health outcomes.

### 4.4. Improve Social Policies and Programs

In the wake of the United States Supreme Court’s decision to overturn Roe v. Wade (Dobbs decision), women of color, communities of low income, and other marginalized populations will be disproportionately impacted by barriers to accessing care [108]. This can lead to increased maternal and infant mortality and an enduring impact on women and families, particularly for Black and rural populations [109]. For example, reduced access to reproductive services could impact high-risk pregnancies. Nationally, Black women are three times more likely to die from a pregnancy-related cause than White women [87]. Another way to address structural racism in birth outcomes through policymaking is to expand access to care in terms of health insurance to include coverage for nonhospital care, doula care, and labor and delivery classes [110]. Policymakers should tackle barriers to doula services that include low reimbursement for Medicaid clients, conflicting certification requirements, and complicated paperwork [111]. There should also be continued Medicaid expansion for postpartum women, including women living in non-expansion states, as timely postpartum care is linked with lower maternal morbidity and mortality [112,113], particularly for Black women [89]. Expanded coverage for behavioral health care should also be considered [88]. There should be an extension of the Medicaid postpartum coverage limit from 60 days (about 2 months) to at least one year [114]. Furthermore, in addition to the need to improve access to reproductive health services, it is imperative to address gaps in maternal support in the U.S., including in the areas of paid family leave, income for women, and child-care affordability [115].

## 5. Conclusions

Pregnancy-related deaths are tragic and mostly preventable. The stark racial disparities in adverse pregnancy outcomes in the U.S. requires a deeper exploration into the role of social determinants and how structural racism contributes to a greater risk of adverse obstetric outcomes among Black women in the U.S. These social determinants include, but are not limited to, neighborhood environments such as access to healthy food, neighborhood safety, housing, air pollution, pest, and mold exposure; environmental exposures including experiences of racism, discrimination, acculturation, and immigration; socioeconomic status factors such as income, education, and occupation; housing conditions; and health care access and quality. Moreover, structural determinants of health such as slavery and structural racism influence social determinants of maternal and infant mortality. The amelioration of these social determinant disparities may also be the answer to decreasing or eliminating the dismal maternal morbidity and mortality rates and may lead to improved health outcomes for Black women in the U.S. Strategies are needed to undo the legacy of racism that fuels unfavorable pregnancy outcomes among Black women in the United States. Recommendations include addressing implicit bias and improving cultural humility in the healthcare sector, diversifying the workforce, incorporating social determinants of health and health disparities into the medical and health professions curriculum, exploring the impact of environmental and occupational exposures on maternal morbidity and mortality, addressing the impact of structural racism on health outcomes, and improving social policies and programs.

## Data Availability

No new data were created or analyzed in this study. Data sharing does not apply to this article.

## References

[1] CDC https://www.cdc.gov/reproductivehealth/maternalinfanthealth/severematernalmorbidity.html.

[2] CDC https://www.cdc.gov/healthequity/features/maternal-mortality/index.html.

[3] CDC https://www.cdc.gov/nchs/data/hestat/maternal-mortality/2020/maternal-mortality-rates-2020.htm.

[4] The American Journal of Managed Care. https://www.ajmc.com/view/us-ranks-worst-in-maternal-care-mortality-compared-with-10-other-developed-nations.

[5] Berg C.J., Callaghan W.M., Syverson C., Henderson Z. (2010). Pregnancy-related mortality in the United States, 1998 to 2005. Obs. Gynecol..

[6] Collins J.W., David R.J., Handler A., Wall S., Andes S. (2004). Very low birthweight in African American infants: The role of maternal exposure to interpersonal racial discrimination. Am. J. Public Health.

[7] Krieger N., Van Wye G., Huynh M., Waterman P.D., Maduro G., Li W., Gwynn R.C., Barbot O., Bassett M.T. (2020). Structural Racism, Historical Redlining, and Risk of Preterm Birth in New York City 2013–2017. Am. J. Public Health.

[8] Thompson T.A.M., Young Y.Y., Bass T.M., Baker S., Njoku O., Norwood J., Simpson M. (2022). Racism Runs Through It: Examining the Sexual And Reproductive Health Experience of Black Women in the South: Study examines the sexual and reproductive health experiences of Black women in the South. Health Aff..

[9] Crear-Perry J., Correa-de-Araujo R., Lewis Johnson T., McLemore M.R., Neilson E., Wallace M. (2021). Social and structural determinants of health inequities in maternal health. J. Women’s Health.

[10] Center for Infectious Disease Research and Policy. https://www.cidrap.umn.edu/news-perspective/2022/06/maternal-deaths-climbed-33-during-covid-19.

[11] Wakeel F., Njoku A. (2021). Application of the Weathering Framework: Intersection of Racism, Stigma, and COVID-19 as a Stressful Life Event among African Americans. Healthcare.

[12] Kotlar B., Gerson E., Petrillo S., Langer A., Tiemeier H. (2021). The impact of the COVID-19 pandemic on maternal and perinatal health: A scoping review. Reprod. Health.

[13] Geronimus A.T. (1992). The weathering hypothesis and the health of African-American women and infants: Evidence and speculations. Ethn. Dis..

[14] NPR https://www.npr.org/sections/codeswitch/2018/91/14/577664626/making-the-case-that-discrimination-is-bad-for-your-health.

[15] Geronimus A.T., Hickens M., Keene D., Bound J. (2006). “Weathering” and Age Patterns of Allostatic Load Scores among Black and Whites in the United States. Am. J. Public. Health.

[16] Medical News Today https://www.medicalnewstoday.com/articles/weathering-what-are-the-effects-of-stress-and-discrimination.

[17] Roach J. (2016). ROOTT’s theoretical framework of the web of causation between structural and social determinants of health and wellness—2016. Restoring Our Own Through Transformation (ROOTT). https://www.roottrj.org/web-causation.

[18] Collins P.H. (2015). Intersectionality’s definitional dilemmas. Annu. Rev. Sociol..

[19] World Health Organization (2022). Social determinants of health. https://www.who.int/health-topics/social-determinants-of-health#tab=tab_1.

[20] World Health Organization (2022). Equity. http://www.who.int/healthsystems/topics/equity/en/.

[21] Alvidrez J., Castille D., Laude-Sharp M., Rosario A., Tabor D. (2019). The national institute on minority health and health disparities research framework. Am. J. Public Health.

[22] (2017). World Health Organization. https://www.who.int/news-room/fact-sheets/detail/human-rights-and-health.

[23] Dagher R.K., Linares D.E. (2022). A Critical Review on the Complex Interplay between Social Determinants of Health and Maternal and Infant Mortality. Children.

[24] Wang E., Glazer K.B., Howell E.A., Janevic T.M. (2020). Social Determinants of Pregnancy-Related Mortality and Morbidity in the United States: A Systematic Review. Obs. Gynecol..

[25] Nelson D.B., Moniz M.H., Davis M.M. (2018). Population-level factors associated with maternal mortality in the United States, 1997–2012. BMC Public Health.

[26] Mehra R., Boyd L.M., Ickovics J.R. (2017). Racial Residential Segregation and Adverse Birth Outcomes: A Systematic Review and Meta-Analysis. Soc. Sci. Med..

[27] Ncube C.N., Enquobahrie D.A., Albert S.M., Herrick A.L., Burke J.G. (2016). Association of Neighborhood Context with Offspring Risk of Preterm Birth and Low Birthweight: A Systematic Review and Meta-Analysis of Population-Based Studies. Soc. Sci. Med..

[28] Howe C.G., Henn B.C., Eckel S.P., Farzan S.F., Grubbs B.H., Chavez T.A., Hodes T.L., Faham D., Al-Marayati L., Lerner D. (2020). Prenatal Metal Mixtures and Birth Weight for Gestational Age in a Predominately Lower-Income Hispanic Pregnancy Cohort in Los Angeles. Environ. Health Perspect..

[29] Owens D.C., Fett S.M. (2019). Black Maternal and Infant Health: Historical Legacies of Slavery. Am. J. Public Health.

[30] Morgan J., Morgan J.L. (2004). Laboring Women: Reproduction and Gender in New World Slavery.

[31] Haines M. Fertility and Mortality in the United States. https://eh.net/encyclopedia/fertility-and-mortality-in-the-united-states/.

[32] Centers for Disease Control and Prevention User guide to the 2016 Period Linked Birth/Infant Death Public Use File. http://ftp.cdc.gov/pub/Health_Statistics/NCHS/Dataset_Documentation/DVS/periodlinked/LinkPE16Guide.pdf.

[33] Miranda M.L., Swamy G.K., Edwards S., Maxson P., Gelfand A., James S. (2010). Disparities in maternal hypertension and pregnancy outcomes: Evidence from North Carolina, 1994–2003. Public Health Rep..

[34] Hicken M.T., Kravitz-Wirtz N., Durkee M., Jackson J.S. (2018). Racial Inequalities in Health: Framing Future Research. Soc. Sci. Med..

[35] Williams D.R., Lawrence J.A., Davis B.A. (2019). Racism and Health: Evidence and Needed Research. Annu. Rev. Public Health.

[36] Johnson T.J. (2020). Intersection of Bias, Structural Racism, and Social Determinants With Health Care Inequities. Pediatrics.

[37] Jones C.P. (2000). Levels of Racism: A Theoretic Framework and a Gardener’s Tale. Am. J. Public Health.

[38] Boyles A.L., Beverly B.E., Fenton S.E., Jackson C.L., Jukic A.M.Z., Sutherland V.L., Baird D.D., Collman G.W., Dixon D., Ferguson K.K. (2021). Environmental factors involved in maternal morbidity and mortality. J. Women’s Health.

[39] Giurgescu C., Misra D.P. (2022). Structural Racism and Maternal Morbidity among Black Women. West. J. Nurs. Res..

[40] Giscombé C.L., Lobel M. (2005). Explaining Disproportionately High Rates of Adverse Birth Outcomes among African Americans: The Impact of Stress, Racism, and Related Factors in Pregnancy. Psychol. Bull..

[41] Mustillo S., Krieger N., Gunderson E.P., Sidney S., McCreath H., Kiefe C.I. (2004). Self-Reported Experiences of Racial Discrimination and Black–White Differences in Preterm and Low-Birthweight Deliveries: The CARDIA Study. Am. J. Public Health.

[42] Rosenberg L., Palmer J.R., Wise L.A., Horton N.J., Corwin M.J. (2002). Perceptions of Racial Discrimination and the Risk of Preterm Birth. Epidemiology.

[43] Witt W.P., Litzelman K., Cheng E.R., Wakeel F., Barker E.S. (2014). Measuring Stress Before and During Pregnancy: A Review of Population-Based Studies of Obstetric Outcomes. Matern. Child Health J..

[44] Bailey Z.D., Krieger N., Agénor M., Graves J., Linos N., Bassett M.T. (2017). Structural racism and health inequities in the USA: Evidence and interventions. Lancet.

[45] Krieger N., Chen J.T., Coull B., Waterman P.D., Beckfield J. (2013). The unique impact of abolition of Jim Crow laws on reducing inequities in infant death rates and implications for choice of comparison groups in analyzing societal determinants of health. Am. J. Public Health.

[46] Gee G.C., Walsemann K.M., Brondolo E. (2012). A life course perspective on how racism may be related to health inequities. Am. J. Public Health.

[47] Humes E. (2006). How the GI Bill Shunted Blacks into Vocational Training. J. Blacks High. Educ..

[48] Alexander M. (2020). The New Jim Crow: Mass Incarceration in the Age of Colorblindness.

[49] Dongarwar D., Ajewole V.B., Oduguwa E., Ngujede A., Harris K., Ofili T.U., Olaleye O.A., Salihu H.M. (2020). Role of Social Determinants of Health in Widening Maternal and Child Health Disparities in the Era of COVID-19 Pandemic. Int. J. MCH AIDS.

[50] Njoku A., Ahmed Y., Bolaji B. (2021). Police brutality against Blacks in the United States and ensuing protests: Implications for social distancing and Black health during COVID-19. J. Hum. Behav. Soc. Environ..

[51] Bray S.R., McLemore M.R. (2021). Demolishing the myth of the default human that is killing Black mothers. Front. Public Health.

[52] CDC https://www.cdc.gov/violenceprevention/about/social-ecologicalmodel.html.

[53] Kaiser Family Foundation https://www.kff.org/report-section/racial-disparities-in-maternal-and-infant-health-an-overview-issue-brief/.

[54] Oribhabor G.I., Nelson M.L., Buchanan-Peart K.A.R., Cancarevic I. (2020). A mother’s cry: A race to eliminate the influence of racial disparities on maternal morbidity and mortality rates among Black women in America. Cureus.

[55] Coussons-Read M.E. (2013). Effects of prenatal stress on pregnancy and human development: Mechanisms and pathways. Obstet. Med..

[56] Jackson F.M., Hogue C.R., Phillips M.T. (2005). The development of a race and genderspecific stress measure for African-American women: Jackson, Hogue, Phillips contextualized stress measure. Ethn. Dis..

[57] March of Dimes https://www.marchofdimes.org/stress-and-pregnancy.aspx.

[58] Jackson F.M., James S.A., Owens T.C., Bryan A.F. (2017). Anticipated Negative Police-Youth Encounters and Depressive Symptoms among Pregnant African American Women: A Brief Report. J. Urban Health.

[59] Courchesne N.S., Smith L.R., Zúñiga M.L., Chambers C.D., Reed M.B., Ballas J., Marienfeld C.B. (2021). Association of alcohol and other substance-related diagnoses with severe maternal morbidity. Alcoholism, clinical and experimental research.

[60] Chakhtoura N., Chinn J.J., Grantz K.L., Eisenberg E., Dickerson S.A., Lamar C., Bianchi D.W. (2019). Importance of research in reducing maternal morbidity and mortality rates. Am. J. Obstet. Gynecol..

[61] Diana S., Wahyuni C.U., Prasetyo B. (2020). Maternal complications and risk factors for mortality. J. Public Health Res..

[62] Tan M., Mamun A., Kitzman H., Mandapati S.R., Dodgen L. (2017). Neighborhood disadvantage and allostatic load in African American women at risk for obesity-related diseases. Prev. Chronic Dis..

[63] Schulz A.J., Mentz G., Lachance L., Johnson J., Gaines C., Israel B.A. (2012). Associations between socioeconomic status and allostatic load: Effects of neighborhood poverty and tests of mediating pathways. Am. J. Public Health.

[64] Cozier Y.C., Albert M.A., Castro-Webb N., Coogan P.F., Ridker P., Kaufman H.W., Palmer J.R., Rosenberg L. (2016). Neighborhood socioeconomic status in relation to serum biomarkers in the Black Women’s Health Study. J. Urban Health.

[65] Robinette J.W., Charles S.T., Almeida D.M., Gruenewald T.L. (2016). Neighborhood features and physiological risk: An examination of allostatic load. Health Place.

[66] O’Campo P., Schetter C.D., Guardino C.M., Vance M.R., Hobel C.J., Ramey S.L., Network C.C.H. (2016). Explaining racial and ethnic inequalities in postpartum allostatic load: Results from a multisite study of low to middle income woment. SSM-Popul. Health.

[67] Howell E.A. (2018). Reducing Disparities in Severe Maternal Morbidity and Mortality. Clin. Obstet. Gynecol..

[68] Mannava P., Durrant K., Fisher J., Chersich M., Luchters S. (2015). Attitudes and behaviours of maternal health care providers in interactions with clients: A systematic review. Glob. Health.

[69] Kumari A., Ranjan P., Sharma K.A., Sahu A., Bharti J., Zangmo R., Bhatla N. (2021). Impact of COVID-19 on psychosocial functioning of peripartum women: A qualitative study comprising focus group discussions and in-depth interviews. Int. J. Gynecol. Obstet..

[70] Singh G.K. (2010). Maternal Mortality in the United States, 1935–2007: Substantial Racial/Ethnic, Socioeconomic, and Geographic Disparities Persist.

[71] Saluja B., Bryant Z. (2021). How Implicit Bias Contributes to Racial Disparities in Maternal Morbidity and Mortality in the United States. J. Women’s Health.

[72] Villarosa L. “Why America’s Black Mothers and Babies Are in a Life-or-Death Crisis,” New York Times Magazine, 11 April 2018. https://www.nytimes.com/2018/04/11/magazine/black-mothers-babies-death-maternal-mortality.html.

[73] Vedam S., Stoll K., Taiwo T.K., Rubashkin N., Cheyney M., Strauss N., Declercq E. (2019). The Giving Voice to Mothers study: Inequity and mistreatment during pregnancy and childbirth in the United States. Reprod. Health.

[74] National Partnership for Women & Families, “Listening to Black Mothers in California,” September 2018. https://www.nationalpartnership.org/our-work/resources/health-care/maternity/listening-to-black-mothers-in-california.pdf.

[75] Lassi Z.S., Kumar R., Bhutta Z.A. (2016). Community-Based Care to Improve Maternal, Newborn, and Child Health. Dis. Control. Priorities.

[76] Mayne S.L., Yellayi D., Pool L.R., Grobman W.A., Kershaw K.N. (2018). Racial residential segregation and hypertensive disorder of pregnancy among women in Chicago: Analysis of electronic health record data. Am. J. Hypertens..

[77] CDC Clinical Tools and Resources. https://www.cdc.gov/hearher/healthcare-providers/clinical-resources-tools.html.

[78] Yusuf K.K., Dongarwar D., Ibrahimi S., Ikedionwu C., Maiyegun S.O., Salihu H.M. (2020). Expected surge in maternal mortality and severe morbidity among African-Americans in the era of COVID-19 pandemic. Int. J. Matern. Child Health AIDS.

[79] Gur R.E., White L.K., Waller R., Barzilay R., Moore T.M., Kornfield S., Elovitz M.A. (2020). The disproportionate burden of the COVID-19 pandemic among pregnant black women. Psychiatry Res..

[80] Shaw E., Levitt C., Wong S., Kaczorowski J., McMaster University Postpartum Research Group (2006). Systematic review of the literature on postpartum care: Effectiveness of postpartum support to improve maternal parenting, mental health, quality of life, and physical health. Birth.

[81] Howell E.A., Egorova N.N., Janevic T., Brodman M., Balbierz A., Zeitlin J., Hebert P.L. (2020). Race and ethnicity, medical insurance, and within-hospital severe maternal morbidity disparities. Obs. Gynecol..

[82] Chan A.L., Guo N., Popat R., Robakis T., Blumenfeld Y.Y., Main E., Scott K.A., Butwick A.J. (2021). Racial and ethnic disparities in hospital-based care associated with postpartum depression. J. Racial. Ethn. Health Disparities..

[83] Brousseau E.C., Danilack V., Cai F., Matteson K.A. (2018). Emergency department visits for postpartum complications. J. Womens Health.

[84] Wilcox A., Levi E.E., Garrett J.M. (2016). Predictors of non-attendance to the postpartum follow-up visit. Matern. Child Health J..

[85] Mi T., Hung P., Li X., McGregor A., He J., Zhou J. (2022). Racial and ethnic disparities in postpartum care in the greater Boston area during the COVID-19 pandemic. JAMA Netw. Open.

[86] Mollard E., Kupzyk K., Moore T. (2021). Postpartum stress and protective factors in women who gave birth in the United States during the COVID-19 pandemic. Women’s Health.

[87] CDC Working Together to Reduce Black Maternal Mortality. https://www.cdc.gov/healthequity/features/maternal-mortality/index.h.

[88] Kaiser Family Foundation https://www.kff.org/racial-equity-and-health-policy/issue-brief/racial-disparities-in-maternal-and-infant-health-current-status-and-efforts-to-address-them/.

[89] Solomon J. Closing the Coverage Gap Would Improve Black Maternal Health. Retrieved from: Penalizing Abortion Providers Will Have Ripple Effects Across Pregnancy Care. https://www.cbpp.org/research/health/closing-the-coverage-gap-would-improve-black-maternal-health.

[90] Bond R., Gaither K., Nasser S., Albert M., Ferdinand K., Njoroge J., Parapid B., Hayes S., Pegus C., Sogade B. (2021). Working Agenda for Black Mothers: A Position Paper from the Association of Black Cardiologists on Solutions to Improving Black Maternal Health. Cardiovasc. Qual. Outcomes.

[91] Lucey C.R., Johnston S.C. (2020). The transformational effects of COVID-19 on medical education. JAMA.

[92] Association of American Medical Colleges https://www.aamc.org/news-insights/medical-students-569%need-learn-about-health-disparities-combat-future-pandemics.

[93] Njoku A., Ammigan R., Chan R., Bista K. (2022). COVID-19 and Health Disparities Opportunities for Public Health Curriculum Enhancement. COVID-19 and Higher Education in the Global Context: Exploring Contemporary Issues and Challenges (pp. 139–153).

[94] Ahn R., Gonzalez G.P., Anderson B., Vladutiu C.J., Fowler E.R., Manning L. (2020). Initiatives to reduce maternal mortality and severe maternal morbidity in the United States: A narrative review. Ann. Intern. Med..

[95] D’Alton M.E., Friedman A.M., Bernstein P.S., Brown H.L., Callaghan W.M., Clark S.L., Grobman W.A., Kilpatrick S.J., O’Keeffe D.F., Montgomery D.M. (2019). Putting the “M” back in maternal-fetal medicine: A 5-year report card on a collaborative effort to address maternal morbidity and mortality in the United States. Am. J. Obstet. Gynecol..

[96] The Commonwealth Fund Policies for Reducing Maternal Morbidity and Mortality and Enhancing Equity in Maternal Health. https://www.commonwealthfund.org/publications/fund-reports/2021/nov/policies-reducing-maternal-morbidity-mortality-enhancing-equity.

[97] Njoku A.U. (2021). COVID-19 and environmental racism: Challenges and recommendations. Eur. J. Environ. Public Health.

[98] Joe J.R., Shillingford-Butler M.A., Oh S. (2019). The Experiences of African American Mothers Raising Sons in the Context of #BlackLivesMatter. TPC.

[99] Malone Gonzalez S. (2019). Making It Home: An Intersectional Analysis of the Police Talk. Gender Soc..

[100] Dreyer B.P. (2021). The Toll of Racism on African American Mothers and Their Infants. JAMA Netw. Open.

[101] Taylor E., Gillborn D., Ladson-Billings G. (2009). Foundations of Critical Race Theory in Education; Critical Educator.

[102] Njoku A., Evans M. (2022). Black women faculty and administrators navigating COVID-19, social unrest, and academia: Challenges and strategies. Int. J. Environ. Res. Public Health.

[103] Yeter D., Banks E.C., Aschner M. (2020). Disparity in Risk Factor Severity for Early Childhood Blood Lead among Predominantly African-American Black Children: The 1999 to 2010 US NHANES. Int. J. Environ. Res. Public Health.

[104] Donley N., Bullard R.D., Economos J., Figueroa I., Lee J., Liebman A.K., Martinez D.N., Shafiei F. (2022). Pesticides and environmental injustice in the USA: Root causes, current regulatory reinforcement and a path forward. BMC Public Health.

[105] Hillier A.E. (2003). Redlining and the home owners’ loan corporation. J. Urban Hist..

[106] Yearby R. (2018). Racial disparities in health status and access to healthcare: The continuation of inequality in the United States due to structural racism: Continuing racial health disparities. Am. J. Econ. Sociol..

[107] Kramer M.R., Hogue C.R. (2009). Is segregation bad for your health?. Epidemiol. Rev..

[108] USA Today https://www.usatoday.com/story/news/health/2022/05/03/people-color-most-impacted-if-roe-v-wade-overturned/9626866002/.

[109] Strasser J., Chen C., Rosenbaum S., Schenk E., Dewhurst E. (2022). Penalizing Abortion Providers Will Have Ripple Effects Across Pregnancy Care. https://www.healthaffairs.org/do/10.1377/forefront.20220503.129912/.

[110] Ogunwole S.M., Bozzi D.G., Bower K.M., Cooper L.A., Hardeman R., Kozhimannil K. (2022). Health Equity Considerations in State Bills Related to Doula Care (2015–2020). Women’s Health Issues.

[111] Van Eijk M.S., Guenther G.A., Kett P.M., Jopson A.D., Frogner B.K., Skillman S.M. (2022). Addressing Systemic Racism in Birth Doula Services to Reduce Health Inequities in the United States. Health Equity.

[112] Kaiser Family Foundation Medicaid Postpartum Coverage Extension Tracker. https://www.kff.org/medicaid/issue-brief/medicaid-postpartum-coverage-extension-tracker/.

[113] Gordon S.H., Sommers B.D., Wilson I.B., Trivedi A.N. (2020). Effects Of Medicaid Expansion On Postpartum Coverage And Outpatient Utilization. Health Aff..

[114] Taylor J.K. (2020). Structural Racism and Maternal Health Among Black Women. J. Law Med. Ethics.

[115] Joyce A., Tierney L. What It’s Like to Have a Baby in the States Most Likely to Ban Abortion. https://www.washingtonpost.com/parenting/2022/05/06/support-in-states-banning-abortion/?utm_source=NIHCM+Foundation&utm_campaign=0ee168971f-nihcm-newsletter-may-2022&utm_medium=email.

